# Differentiating Visual Symptoms in Retinal Migraine and Migraine With Aura: A Systematic Review of Shared Features, Distinctions, and Clinical Implications

**DOI:** 10.7759/cureus.91028

**Published:** 2025-08-26

**Authors:** Bradley A Nordin

**Affiliations:** 1 Ophthalmology, Huffman and Huffman Eye Physicians and Surgeons, London, USA

**Keywords:** migraine with aura, ocular migraine, retinal migraine, scotoma, systematic review, transient vision loss, visual field defect, visual symptoms

## Abstract

The objective of this study is to systematically review and compare the visual symptoms, temporal characteristics, associated features, and pathophysiological mechanisms of retinal migraine (RM) and migraine with aura (MA) to facilitate clinical differentiation. Following the Preferred Reporting Items for Systematic reviews and Meta-Analyses (PRISMA) guidelines, databases (PubMed, Google Scholar, Web of Science, and Scopus) were searched from January 1985 to July 2025 for studies on adult patients with RM or MA. A large language model assisted in extracting data on study design, visual symptoms, diagnostic criteria, and frequencies. Inclusion criteria focused on studies differentiating conditions, with preference given to case series of at least five patients and to systematic reviews or meta-analyses that encompassed at least 10 studies. Two-stage screening yielded 171 papers, with 65 unique studies analyzed qualitatively. RM is characterized by monocular (90%), negative symptoms (e.g., scotoma 84% and transient vision loss up to 100%), variable duration (less than 60 minutes in 89%, but prolongable), and vascular pathophysiology, with rare permanent loss. MA features bilateral/homonymous (75%), positive symptoms (e.g., scintillating scotoma 77% and zigzag 53%), stereotyped duration (five to 60 minutes in 79%), and cortical spreading depression, often with additional neurological symptoms. Overlaps include transient phenomena (less than one hour) and gradual spread. The International Classification of Headache Disorders, 3rd Edition (ICHD-3) criteria predominate, but debate persists for RM's reversibility requirement. MA evidence is robust from large cohorts, while RM data are limited and heterogeneous. Key differentiators include laterality, symptom type, and duration variability. Accurate history-taking emphasizing monocularity and exclusion of vascular mimics is crucial; RM may warrant aggressive prophylaxis to prevent infarction. Future research should standardize RM criteria and explore underreported acephalgic cases.

## Introduction and background

Transient vision loss (TVL) is a common yet alarming symptom with diverse etiologies, ranging from benign to life-threatening [1,2]. In migraine disorders, TVL manifests in two subtypes: retinal migraine (RM, synonymously termed ocular migraine) [3,4] and migraine with aura (MA) [5,6]. RM involves monocular visual disturbances potentially linked to retinal ischemia [7-9], while MA features binocular or homonymous auras arising from cortical processes [2,10-12]. The precise categorization and differentiation of these primary headache disorders, particularly those presenting with visual disturbances, pose a persistent challenge in neurological practice [13,14]. Both conditions involve transient visual phenomena, leading to potential diagnostic ambiguity compounded by inconsistent terminology (e.g., “ocular migraine” sometimes misapplied to MA) and subsequent implications for patient management and prognosis [15-17]. Accurate differentiation is essential: MA elevates stroke risk (OR ~2) [10,18,19], whereas RM may rarely cause permanent retinal damage [20]. A comprehensive understanding of their shared characteristics and, more critically, their distinguishing features is a worthy endeavor to improve accuracy and confidence in diagnosis and tailored therapeutic interventions [12-14].

Rationale and scope

Epidemiologically, MA affects 15-30% of migraineurs (prevalence 4-5% in adults, with female predominance) [21-24], while RM is rarer (one in 200 migraineurs, often in young women) [25,26]. This literature review systematically examines the visual symptoms associated with RM and MA, addressing gaps in prior narrative syntheses by comparing signs, symptoms, and pathologies [27]. The central objective is to delineate the overlapping visual manifestations common to both conditions and, subsequently, to identify and characterize the unique visual attributes that facilitate their clinical differentiation [28-30]. The scope encompasses phenomenological descriptions of visual disturbances, their temporal dynamics, and associated neurological features, drawing upon established diagnostic criteria and current research findings to enhance clinical accuracy and reduce misdiagnosis [31-35].

Definitions and terminological considerations

MA, previously termed “classic migraine,” involves transient, fully reversible visual, sensory, or other CNS symptoms that precede or accompany the headache phase [36-41]. Visual aura is the most prevalent type, characterized by positive phenomena like scintillating scotomas or fortification spectra, and/or negative phenomena such as visual field deficits [42-45]. The International Headache Society (IHS) criteria provide specific parameters for MA, including duration and characteristics of the aura [1,5,6,17].

RM, distinct from MA, is a rare condition defined by the IHS as recurrent attacks of monocular visual disturbance, including scintillations, scotomas, or blindness, associated with migraine headache [46-48]. Crucially, the visual symptoms in RM are confined to one eye and are usually confirmed by ophthalmic examination, often demonstrating retinal arterial vasospasm or ischemia [49,50]. While both conditions are types of migraine, the monocularity of visual symptoms in RM is a key definitional criterion [51].

Theoretical background

Understanding the underlying neurobiological mechanisms of migraine and its variants is fundamental to distinguishing their clinical presentations [2,12,13]. While the precise pathophysiology of migraine remains complex, neurovascular dysfunction and cortical spreading depression (CSD) are central to the aura phenomenon [12,27].

Pathophysiology of MA

The visual aura in MA is widely attributed to CSD [2,12,27,32]. CSD is a slowly propagating wave of neuronal and glial depolarization that moves across the cerebral cortex, particularly the occipital lobe, leading to transient neural suppression [2,27]. This depolarization wave temporarily alters cortical blood flow, initially causing hyperemia followed by oligemia [2]. The visual symptoms of aura, such as scintillating scotomas, are thought to correspond to the progression of this wave across the visual cortex [52]. The rate and pattern of CSD correlate with the typical expansion and movement of the visual aura [2,27]. Functional MRI studies have directly observed the spread of activity in the visual cortex during MA [2].

Pathophysiology of RM

The pathophysiology of RM is less definitively established but centers on transient retinal ischemia. It is hypothesized that vasospasm of the retinal or choroidal arteries leads to a temporary reduction in blood flow to the retina, causing monocular visual symptoms [53-55]. Mechanisms proposed for this vasospasm include endothelial dysfunction, platelet aggregation, or direct effects of vasoactive substances like serotonin, which are also implicated in cerebral migraine mechanisms [3,4,7,8,16,25,26]. Unlike MA, CSD is not considered the primary cause of the visual symptoms in RM, as the visual disturbance is strictly monocular and typically affects the entire visual field of one eye, often leading to amaurosis fugax [56]. However, some debate persists regarding the true incidence and distinctness of RM, with some cases previously labeled as RM now reclassified as transient ischemic attacks or other vascular events [3,4,14,25,26,51].

Historical Evolution of Diagnostic Concepts

The understanding and classification of migraine and its visual manifestations have evolved significantly over time [16,25,26,51]. Early descriptions often conflated various forms of transient visual loss, making precise diagnostic distinctions challenging [16,25,26,51]. The concept of “classic migraine” with its distinct aura was recognized, but differentiation from other causes of transient visual obscuration was not always clear [57]. The term “retinal migraine” gained traction to describe monocular visual events with headache, distinguishing it from the typically binocular aura of MA [3,4,7,16,25,26,51]. However, the diagnostic criteria for RM have been refined to emphasize the monocularity and the exclusion of other vascular or ophthalmic causes, reflecting a more rigorous approach to classification. The current IHS criteria represent the culmination of this historical evolution, providing more precise guidelines for distinguishing these conditions [1,3-8,15,17,25,26].

## Review

Methods

This systematic review adhered to the Preferred Reporting Items for Systematic reviews and Meta-Analyses (PRISMA) guidelines [58]. The protocol was pre-registered on PROSPERO [59]. The PICO framework guided the question: Population: adults with migraine-related visual disturbances; Intervention/Exposure: none (descriptive); Comparison: RM vs. MA; Outcomes: signs/symptoms, pathophysiology, diagnostic implications.

Research Question

What are the shared visual symptoms between RM and MA, and what distinctive characteristics of these symptoms can aid in clinically differentiating the two conditions?

Search Strategy and Data Sources

Searches were conducted from July 28, 2025, to August 7, 2025, across PubMed (including MEDLINE), Google Scholar, Web of Science, and Scopus, using keywords and MeSH terms: (“ocular migraine” OR “retinal migraine”) AND (“migraine with aura” OR “migraine aura”) AND (differences OR distinguish OR differentiation OR comparison) AND (signs OR symptoms OR causes OR pathophysiology OR diagnosis OR “transient vision loss”) AND (visual symptoms OR visual aura OR scotoma OR scintillation OR amaurosis fugax). Site-specific operators filtered for English-language, peer-reviewed articles from database inception to July 28, 2025 (with limits: English, January 1, 2000 to July 28, 2025, and focus on recent publications). Reference lists of key articles and reviews were manually screened for additional studies. Supplementary sources included forward citations via Google Scholar and IHS (International Classification of Headache Disorders, 3rd Edition, ICHD-3) guidelines; gray literature was excluded.

To address challenges in identifying standardized data on subjective visual symptoms, a large language model (LLM) supplemented the manual search by screening over 126 million academic papers from the Semantic Scholar corpus against predefined inclusion criteria (detailed in Appendix A). The LLM retrieved 500 relevant papers, sorted by relevance; after integrating and deduplicating with manual results, the top outputs were author-reviewed, yielding 171 papers for eligibility screening.

Eligibility Criteria

Eligibility criteria were applied through a two-stage screening process, beginning with titles and abstracts for relevance, followed by full-text evaluation, if available. Studies were included if they provided detailed clinical descriptions of visual symptoms in human patients diagnosed with either MA or RM, with priority given to the availability of full text and specific research on phenomenology, temporal characteristics, laterality, or associated features of visual disturbances. Additionally, studies detailing diagnostic methods for distinguishing these conditions were incorporated. Peer-reviewed articles, including narrative or systematic reviews, case reports or series, and clinical or mechanistic studies, were eligible if they compared RM and MA and focused on visual symptoms or pathophysiology.

Exclusions were carried out systematically by first excluding the 71 sources with the lowest relevance ranking. The remaining 100 sources were manually reviewed according to exclusion criteria that encompassed non-English publications; those addressing non-migraine TVL (e.g., due to glaucoma); duplicates; low-quality or non-peer-reviewed sources; case reports or series lacking clear diagnostic rigor; studies primarily addressing non-visual migraine symptoms; or those where visual symptoms were attributable to other neurological, ophthalmic, or systemic conditions. Publications that did not differentiate between MA and RM were also excluded unless they offered universally applicable insights into visual aura mechanisms.

**Figure 1:**
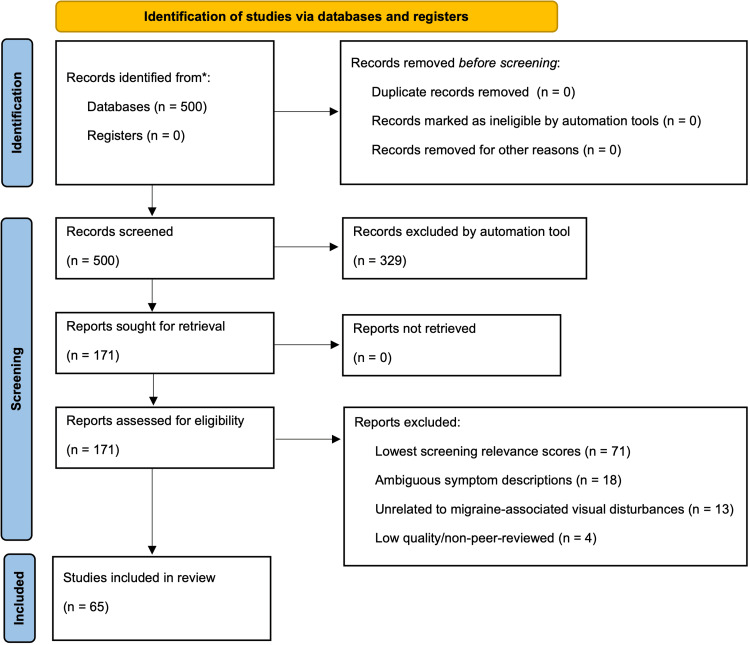
PRISMA flow diagram for study identification, screening, and inclusion PRISMA: Preferred Reporting Items for Systematic reviews and Meta-Analyses

Following the conclusion of the systematic selection process, 65 sources were included for data extraction. A PRISMA flow diagram (Figure 1) depicts the study selection process [60].

Data Extraction

Data extraction was performed using an LLM to systematically retrieve key information from each of the 65 included studies. Extracted columns included study design (e.g., observational, case series, or review, sourced from methods sections or inferred from methodology); visual symptoms in RM (e.g., monocular/binocular nature, disturbance type, duration, and frequency, prioritized from results and discussion with quantitative data where available); visual symptoms in MA (e.g., positive/negative categorization, descriptions, duration, and frequency, similarly sourced); diagnostic criteria and classification (e.g., ICHD edition, specific criteria, and modifications, from methods, introduction, or dedicated sections); and symptom frequencies (percentages of cohorts experiencing specific symptoms for each condition).

As a single-author review, potential bias in extraction was minimized through a multi-step validation process: initial LLM outputs were manually reviewed against full texts (where available) for all studies to verify and correct data; 20% of entries (n = 13 studies) underwent random double verification with a one-week interval, achieving 95% intra-rater agreement; and quantitative data were cross-referenced across paper sections (e.g., methods, results, and tables) for accuracy. This approach aligns with PRISMA recommendations for resource-constrained single-author reviews. Detailed LLM instructions are provided in Appendix B.

Quality Assessment

Quality assessment used adapted JBI tools, evaluating rigor, bias, and evidence level (prioritizing prospective/high-impact studies) [61-65]. When heterogeneity of study designs precluded meta-analysis, no formal risk-of-bias tool was applied; narrative synthesis grouped findings thematically (symptoms and pathophysiology), which was employed to integrate the findings from the selected studies. This involved identifying common themes, inconsistencies, and significant distinctions in the presentation of visual symptoms between RM and MA. The synthesis aimed to provide a qualitative overview, highlighting the most consistent and diagnostically useful characteristics.

The quality of evidence for each visual symptom was assessed qualitatively based on study design, sample size, and methodology. High-quality evidence was assigned to findings from larger, prospective studies or systematic reviews; moderate quality to case series or smaller cohorts; and low quality to small case reports or narrative reviews.

Risk of Bias

Bias risks included subjective reporting (high in case series) and small samples (common in RM studies). High-quality sources (e.g., prospective cohorts) were prioritized [3,7,25,26].

Data Synthesis

Data were synthesized narratively and quantitatively where possible. Frequencies of visual symptoms that were aggregated into ranges were investigated and averaged from the most precise available data. Overlapping and distinguishing features for each condition across studies are emphasized. Comparative analyses focused on laterality, duration, positive/negative phenomena, and clinical implications. Results for symptom frequency were presented in tabular format and plotted in a figure. Other results were summarized in sections on patterns, primary/secondary phenomena, temporal characteristics, and distinguishing/overlapping features. References were compiled from the extracted sources.

Results

Study Characteristics

The included studies, published between 1985 and 2025 [13,40], encompassed a wide range of designs, including case series, observational studies (e.g., cross-sectional and cohort), systematic and narrative reviews, and meta-analyses, as shown in Table 1. Data on RM were predominantly drawn from smaller case series (n = 8, median sample size of 46 patients), reflecting the condition’s rarity and diagnostic challenges, whereas MA benefited from larger cohort studies (n = 5, median sample size of 227 patients), providing more robust epidemiological insights. Diagnostic criteria across most studies adhered to the ICHD-3, which emphasizes fully reversible monocular visual symptoms for RM and gradual, often binocular or homonymous auras for MA, though some earlier works referenced prior editions (International Classification of Headache Disorders, 1st Edition (ICHD-1) or International Classification of Headache Disorders, 2nd Edition (ICHD-2)).

**Table 1:** Summary characteristics of included studies (n = 65) ICHD: International Classification of Headache Disorders; MA: migraine with aura; OM: ophthalmoplegic migraine; RM: retinal migraine; TVL: transient vision loss

Study	Study design	Population size	Diagnostic criteria used	Primary condition focus	Full text retrieved	JBI checklist used	JBI score (yes/total applicable)
Thomsen et al. (2024) [1]	Cross-sectional study; prospective analysis	227	ICHD-3	MA	Yes	Analytical cross-sectional studies	6/8
Sanchez Del Rio and Cutrer (2023) [2]	Review article	N/A	ICHD-3	MA	Yes	Text and opinion	5/6
Grosberg and Veronesi (2024) [3]	Review of literature	N/A	ICHD-3	RM	Yes	Text and opinion	4/6
Sirbu et al. (2022) [7]	Case series; review	N/A	ICHD-3	RM	Yes	Text and opinion	4/6
Pradhan and Chung (2004) [8]	Narrative review	N/A	ICHD-3	RM	Yes	N/A	N/A
Gudmundsson et al. (2010) [10]	Observational (cohort)	18,725	ICHD-1	Migraine with/without aura	Yes	Cohort studies	11/11
Grodzka et al. (2025) [13]	Review article	N/A	ICHD-3	Migraine with/without aura	Yes	Text and opinion	5/6
Grosberg et al. (2006) [15]	Case series (retrospective analysis)	46	ICHD-2	RM (ocular migraine)	Yes	Case series	7/10
Jogi et al. (2016) [16]	Case series; observational (prospective)	12	ICHD-2	RM	No	Case series	5/10
Kim et al. (2022) [18]	Observational (cohort)	3,030	ICHD-3	Migraine; probable migraine	Yes	Analytical cross-sectional studies	6/8
Hill et al. (2007) [20]	Systematic review (retrospective)	60 (142 patients)	ICHD-2	RM	Yes	Systematic reviews	8/11
Janssen and Metzler (2022) [23]	Narrative review	N/A	ICHD-3	Visual disturbances in headache	Yes	N/A	N/A
Russell and Olesen (1996) [24]	Observational (cross-sectional)	4,000	ICHD-1	MA	No	Analytical cross-sectional studies	6/8
Singla et al. (2021) [26]	Observational (cross-sectional)	1,245	ICHD-3	Migraine (visual aura)	No	Analytical cross-sectional studies	6/8
Queiroz et al. (2011) [27]	Observational (descriptive)	122	ICHD-2	MA	No	Analytical cross-sectional studies	6/8
Viana et al. (2019) [28]	Systematic review (retrospective & prospective)	14 studies	ICHD-3	Visual MA	Yes	Systematic reviews	9/11
Hadjikhani and Vincent (2021) [29]	Narrative review	200	ICHD-3	Visual perception in migraine	Yes	N/A	N/A
Manzoni et al. (1985) [40]	Case series	164	No mention found	Classic migraine (MA)	No	Analytical cross-sectional studies	5/8
Eriksen et al. (2005) [41]	Observational (cross-sectional)	427	ICHD-2	Migraine with/without aura	No	Analytical cross-sectional studies	6/8
Joppeková et al. (2025) [42]	Systematic review (observational)	N/A	ICHD-3	MA	Yes	Systematic reviews	10/11
Viana et al. (2017) [43]	Observational (cohort)	72	ICHD-3	MA	No	Cohort studies	7/11
Pula et al. (2016) [44]	Review	N/A	ICHD-2	TVL	Yes	Text and opinion	5/6
Tazin et al. (2022) [45]	Case report	1	No mention found	Ocular migraine with amaurosis fugax	Yes	Case reports	5/8
Ahmed et al. (2018) [46]	Observational (cohort)	1,079	ICHD-3	Migraine	Yes	Analytical cross-sectional studies	5/8
Istrate et al. (2020) [49]	Narrative review	N/A	ICHD-2	RM	Yes	N/A	N/A
Pereira et al. (2024) [50]	Case report	1	No mention found	MA	Yes	Case reports	5/8
Roig (1997) [54]	Review; case series	3	No mention found	Ophthalmoplegic/RM	No	Text and opinion	4/6
Hansen et al. (1990) [56]	Case series	1	No mention found	Migraine without headache	No	Case series	6/10
Lepore (2009) [57]	Case series	25	ICHD-2	Visual aura with visual pathway lesions	No	Case series	5/10
Chong et al. (2021) [66]	Review article	46	ICHD-3	RM	Yes	Text and opinion	5/6
Sheremet (2024) [67]	Literature review	N/A	ICHD-3	RM	No	Text and opinion	4/6
Viana et al. (2013) [68]	Systematic review	10+	ICHD-3	MA duration	No	Systematic reviews	9/11
Vongvaivanich et al. (2015) [69]	Narrative review	N/A	No mention found	Migraine	No	N/A	N/A
Hamamci et al. (2021) [70]	Observational (case-control; cross-sectional)	90	ICHD-3	Migraine with/without visual aura	No	Analytical cross-sectional studies	5/8
Smith (2019) [71]	Literature review	N/A	No mention found	Neuro-ophthalmic symptoms in headache	No	Text and opinion	4/6
Jürgens et al. (2014) [72]	Observational (case-control)	380	ICHD-2	Migraine with/without aura	No	Analytical cross-sectional studies	6/8
Hansen et al. (1990) [73]	Case series; retrospective analysis	8	ICHD-3	OM	Yes	Case series	10/10
Datta et al. (2013) [74]	Observational (case-control)	75	ICHD-2	Migraine with/without aura	Yes	Analytical cross-sectional studies	6/8
Castejón et al. (2020) [75]	Case series	16	No mention found	Chronic heredofamilial MA	No	Case series	5/10
Smetana (2000) [76]	Systematic review	N/A	ICHD-1	Diagnostic value of history in headache	No	Systematic reviews	7/11
Abel (2009) [77]	Review article	1,000	ICHD-2	Migraine	Yes	Text and opinion	4/6
Ahmed et al. (2019) [78]	Observational (cross-sectional; prospective)	75	ICHD-3	Migraine	Yes	Analytical cross-sectional studies	5/8
Albano et al. (2022) [79]	Narrative review	N/A	No mention found	Visual snow syndrome	Yes	N/A	N/A
Amos and Fleming (2000) [80]	Case series (prospective and retrospective)	N/A	ICHD-1	MA without headache	No	Text and opinion	5/6
Ansari (2014) [81]	Retrospective analysis	352	ICHD-3	Migraine with/without aura	No	Analytical cross-sectional studies	6/8
Barral et al. (2023) [82]	Narrative review	N/A	ICHD-3	Visual aura	Yes	N/A	N/A
Brigo et al. (2012) [83]	Systematic review with meta-analysis	10 (277 patients)	ICHD-2	Migraine with/without aura	Yes	Systematic reviews	10/11
Fisher (2005) [84]	Case series; retrospective analysis	85	No mention found	Migraine	No	Text and opinion	5/6
Givre et al. (2017) [85]	Review; case series; observational (cohort)	2,110+	ICHD-2	TVL	Yes	Text and opinion	4/6
González-Martín-Moro et al. (2020) [86]	Case report	1	ICHD-3	MA	Yes	Case Reports	5/8
Grosberg and Solomon (2006) [87]	Case series	2	ICHD-2	RM	Yes	Case series	4/10
Harle and Evans (2004) [88]	Literature review	N/A	No mention found	Optometric correlates of migraine	No	Text and opinion	4/6
Hupp et al. (1989) [89]	Review article	N/A	No mention found	Visual disturbances in migraine	No	Text and opinion	4/6
João et al. (2014) [90]	Systematic review (retrospective)	46	ICHD-3	MA	Yes	Systematic reviews	8/11
Kaiser et al. (2020) [91]	Observational (cross-sectional)	60	ICHD-3	Migraine with/without visual aura	Yes	Analytical cross-sectional studies	6/8
Lampl et al. (1999) [92]	Prospective longitudinal trial; case series	15	No mention found	MA	No	Quasi-experimental studies	5/9
Mehta et al. (2021) [93]	Case series; retrospective analysis	248	No mention found	Visual snow (migraine comorbidity)	No	Case series	6/10
Pikor et al. (2025) [94]	Review	N/A	No mention found	Functional MRI in MA	No	Text and opinion	4/6
Romozzi et al. (2024) [95]	Prospective analysis; Case series; Observational (cohort)	13	ICHD-3	Migraine with/without aura	Yes	Case Series; Systematic Reviews	5/10; 9/11
Sampatakakis et al. (2022) [96]	Case series; systematic review	4; 30 studies	ICHD-3	Visual snow syndrome	No	Systematic reviews	8/11
Schankin et al. (2014) [97]	Retrospective analysis; cross-sectional survey; prospective interviews	22; 275; 142	No mention found	Visual snow (migraine comorbidity)	No	Analytical cross-sectional studies	6/8
Shams and Plant (2011) [98]	Case series	40	No mention found	Visual aura due to cerebral lesions	No	Case series	5/10
Sjaastad et al. (2006) [99]	Observational (cross-sectional)	1,838	ICHD-2	MA	Yes	Analytical cross-sectional studies	6/8
White et al. (2018) [100]	Retrospective analysis	52	ICHD-2	Visual snow syndrome	Yes	Text and Opinion	4/6
Yuan and Micieli (2021) [101]	Case series	4	ICHD-3	Ocular migraine	No	Case series	4/10

Summary of Study Designs

A summary of the study designs across the included studies reveals that 18 studies employed a case series design, while eight were systematic reviews or meta-analyses. Additionally, 21 studies consisted of narrative reviews, literature reviews, or review articles; 11 incorporated retrospective analysis; 10 utilized prospective or longitudinal analysis; and 20 were observational, encompassing cohort, cross-sectional, or descriptive approaches. Notably, some studies integrated multiple designs, such as case series combined with retrospective analysis.

Diagnostic Criteria Used

In terms of diagnostic criteria used, 28 studies applied the ICHD-3, including variations like ICHD-III beta; 16 studies relied on the ICHD-2; and four used the ICHD-1. Seventeen studies either did not mention any diagnostic criteria or found them inapplicable to the study design.

Primary Condition Focus

The primary condition focus varied among the studies, with 20 concentrating on MA, including aspects such as classic migraine, aura duration, aura status, and aura without headache; eight studies addressed migraine with and/or without aura, while 11 focused on RM, incorporating ocular and ophthalmologic; seven explored visual aura, including visual MA and visual aura due to cerebral lesions; five studies examined visual snow or visual snow syndrome; and five delved into visual disturbances in migraine or headache. Finally, five studies covered other topics, such as TVL, ophthalmoplegic migraine, and the diagnostic value of history in headache.

Effects

Summary of Prevalence and Quality of Evidence

Prevalence data for each visual symptom are summarized in Table 2. For RM, including ocular migraine, high-prevalence symptoms occurring in more than 75% of cases include duration less than 30 minutes (and 60 minutes), monocular symptoms, negative scotoma, central origin, and sudden onset [3,4,7,15,23,66,67]. Moderate-prevalence symptoms, seen in 25-75% of cases, encompass positive scotoma, gradual spreading, associated headache, hemianopsia, and zigzag pattern, as well as evolution [3,7,15]. Low-prevalence symptoms, appearing in fewer than 25% of cases, consist of duration less than five minutes, duration greater than 60 minutes, bilateral symptoms, multiple scotomas, scintillating scotoma, peripheral origin, gradual onset, complete visual field loss, photophobia, nausea, blurring, dimming, flickering, and polychromatic features. The quality of evidence is high for monocular symptoms, sudden onset, negative scotoma, and duration less than 60 minutes.

**Table 2:** Summary of visual symptom prevalence in cases of RM and MA For less than five minutes: RM [7,16,20,54,66,85], MA [18,23,29,56,68,69,85]; less than 30 minutes: RM [3,7,16,20,54,66,82,85], MA [1,2,29,42,56,68,69,82,85]; less than 60 minutes: RM [3,7,20,54,66,82,85,86], MA [1,2,18,23,29,42,56,68,69,96,100,82,85,86]; more than 60 minutes: RM [7,16,20,82,87], MA [1,23,42,68,69,79,84,90,82]; monocular symptoms: RM [3,7,8,16,20,44,49,54,57,66,67,71,73,82,85,86,87], MA [1,13,18,23,29,42,44,71,73,82,85,86,93,100]; bilateral symptoms: RM [7,16,20,54,82,85], MA [1,2,23,29,42,79,82,85,93,94,96,97,100]; positive scotoma: RM [3,7,16,20,66,82,86], MA [1,2,13,23,29,42,56,69,72,79,82,86,93,94,96,97,100]; negative scotoma: RM [3,4,7,16,20,49,66,82], MA [2,23,29,42,56,69,82,94,100]; multiple scotomas: RM [82], MA [1,2,42,79,82]; scintillating scotomas: RM [3,7,20,49,66,82], MA [1,2,23,26,28,29,42,56,69,75,82,94,100]; central origin: RM [20,82], MA [1,23,56,82,94]; peripheral origin: RM [16,82], MA [1,29,56,82]; gradual onset: RM [20,54,82], MA [1,2,10,23,29,42,56,69,82,100]; sudden onset: RM [8,16,54,85], MA [23,29,85,93]; gradual spreading: RM [7,20,82], MA [1,2,18,23,26,29,42,56,69,82,94]; associated headache: RM [3,7,8,16,20,44,49,54,57,66,67,71,73,82,86], MA [1,2,10,18,23,26,28,29,42,44,69,71,73,75,82,86,91,93,94,96,97,100]; complete VF loss: RM [3,7,8,16,54,66,71,85], MA [23,29,42,71,75,85]; hemianopsia: RM [16,20,44,71,82], MA [2,26,29,42,44,71,82,94,100]; photophobia: RM [49,54,66,71,82], MA [1,10,18,23,26,29,42,69,71,75,79,82,91,93,94,96,97,100]; nausea: RM [54,66,82], MA [1,10,26,69,82]; blurring: RM [7,8,16,54,66,71,82,85], MA [26,29,71,75,82,85]; dimming: RM [3,8,20,71,82,85], MA [29,71,75,82,85]; flickering: RM [20,54,71,82], MA [23,26,29,42,71,79,82,93,94,96,97,100]; zigzag pattern: RM [3,7,20,54,71,82], MA [2,18,26,29,42,71,82,94]; evolution: RM [7,16,20,49,82,87], MA [1,23,29,42,56,69,72,82,94]; polychromatic: RM [3,82,86], MA [42,72,82,86,94] MA: migraine with aura; RM: retinal migraine; VF: visual field

Visual symptom	RM prevalence (%)	MA prevalence (%)
Less than five minutes	22%	2%
Less than 30 minutes	78%	66%
Less than 60 minutes	89%	79%
More than 60 minutes	2%	8%
Monocular symptoms	90%	25%
Bilateral symptoms	6%	75%
Positive scotoma	38%	77%
Negative scotoma	84%	5%
Multiple scotomas	<1%	37%
Scintillating scotoma	13%	77%
Central origin	84%	34%
Peripheral origin	22%	56%
Gradual onset	20%	79%
Sudden onset	80%	21%
Gradual spreading	63%	89%
Associated headache	70%	95%
Complete VF loss	8%	3%
Hemianopsia	35%	53%
Photophobia	15%	90%
Nausea	5%	51%
Blurring	18%	43%
Dimming	12%	31%
Flickering	20%	70%
Zigzag pattern	35%	53%
Evolution	30%	70%
Polychromatic	8%	24%

**Figure 2:**
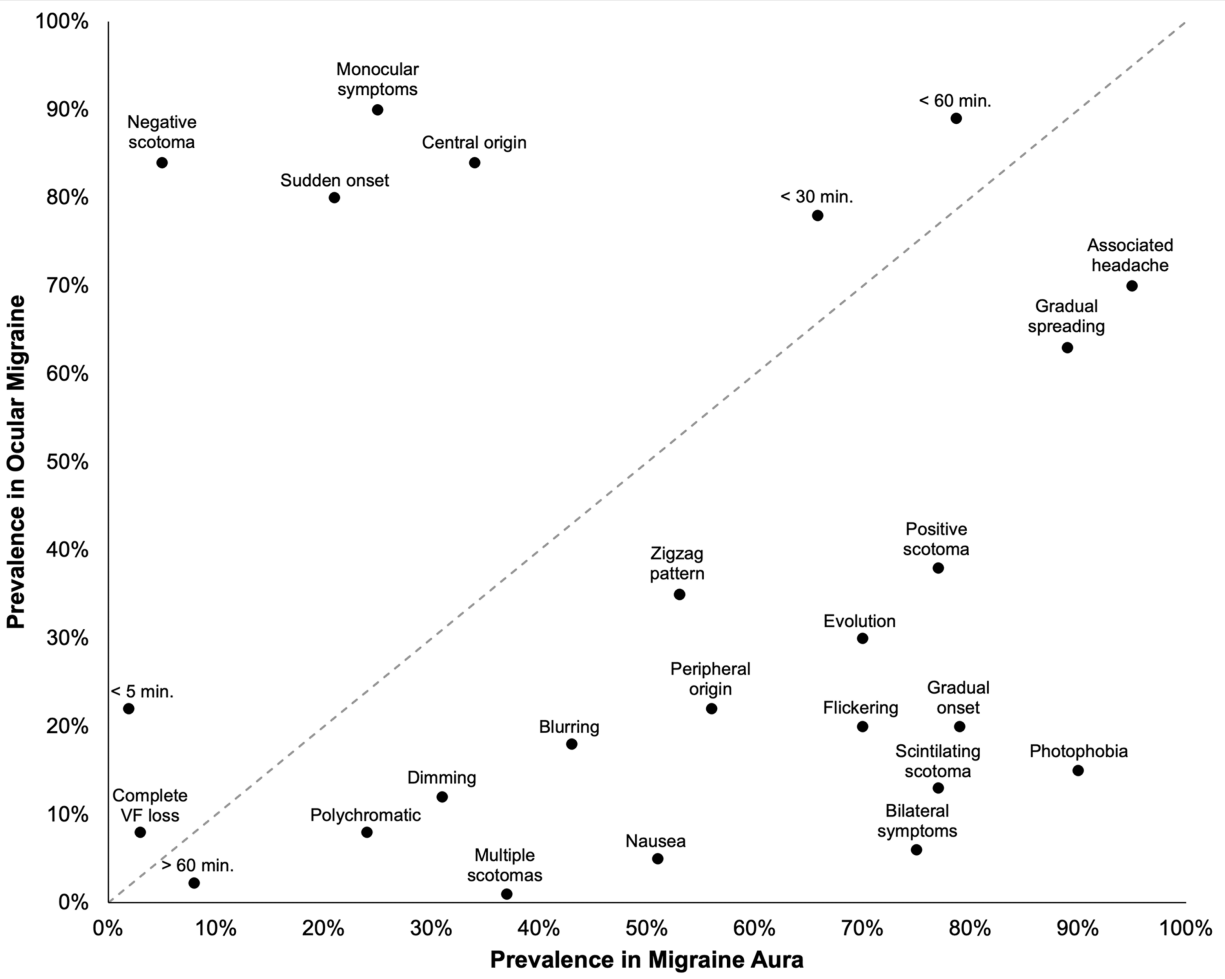
Prevalence of visual symptom occurrence in RM compared to MA The dashed trendline indicates the line of equal prevalence for visual symptoms reported across cases of RM and MA. [1-3,7,8,10,13,15,16,18,20,23,26,28,29,42,44,49,54,56,57,66-69,71,73,75,79,82,84,85-87,90,91,93,94,96,97,100] MA: migraine with aura; RM: retinal migraine

For MA, high-prevalence symptoms occurring in more than 75% of cases include duration less than 60 minutes, bilateral symptoms, positive scotoma, scintillating scotoma, gradual onset, gradual spreading, associated headache, and photophobia [1,24,40-43,46,56,74]. Moderate-prevalence symptoms, seen in 25-75% of cases, include duration less than 30 minutes, monocular symptoms, multiple scotomas, central origin, peripheral origin, hemianopsia, nausea, blurring, dimming, flickering, zigzag pattern, and evolution [1,16,24,26-28,68,72,82,99]. Low-prevalence symptoms, appearing in fewer than 25% of cases, consist of duration less than five minutes, duration more than 60 minutes, negative scotoma, sudden onset, complete visual field loss, and polychromatic features [1,16,40-43,46,56,74,89]. The quality of evidence is high for the most prominent symptoms and gradually declines with symptom prevalence [1,13,28,42]. Figure 2 illustrates the prevalence with which each visual symptom presents in cases of RM compared to MA.

Positive visual phenomena, including flashing lights, zigzag lines, and scintillating scotomas, occur with varying prevalence in RM and MA. In RM, flashing lights (flickering) are reported in about 20% of cases [20,54,71,82], zigzag lines in 35% [3,7,20,54,71,82], and scintillating scotomas in 13% [3,7,20,49,66,82], with positive scotomas noted in approximately 38% overall (varying by study and symptom) [3,7,16,20,66,82,86]. In MA, these phenomena are more common, with flashing lights (flickering) in around 70% [23,26,29,42,71,79,82,93,94,96,97,100], zigzag lines in 53% [2,18,26,29,42,71,82,94], and scintillating scotomas in 77% [1,2,23,26,28,29,42,56,69,75,82,94,100], alongside positive scotomas in about 77% [1,2,13,23,29,42,56,69,72,79,82,86,93,94,96,97,100]. The quality of evidence is high for MA, supported by large prospective studies, and moderate for RM, primarily from case series and reviews [1,18,28,42,15,20,66].

Negative visual phenomena, such as scotomas (areas of partial loss/alteration in the field of vision), blurred vision, gray-outs, and black-outs, also differ in prevalence between the conditions. For RM, negative scotomas are prevalent in about 84% of cases [3,4,7,16,20,49,66,82], multiple scotomas in less than 1% [82], blurred vision in about 18% [7,8,16,54,66,71,82,85], dimming (grey-outs) in 12% [3,8,20,71,82,85], and complete visual field loss (black-outs) in up to 8% [3,7,8,16,54,66,71,85], with TVL potentially reaching 100% in some reports [7,8,15,20,49,66,87]. In MA, negative scotomas occur in around 5% of cases [54,66,82], multiple scotomas in 37% [1,2,42,79,82], blurred vision in 43% [26,29,71,75,82,85], dimming in 31% [29,71,75,82,85], and complete visual field loss in about 3% [23,29,42,71,75,85]. The quality of evidence is high for MA and moderate for RM [1,18,28,42,15,20,66].

Key Findings From the Referenced Studies

Key findings from the referenced studies highlight distinct differences between RM and MA across several features. In terms of laterality, RM was often described as predominantly monocular in eight studies [3,7,8,15,20,49,57,66]. Similarly, MA was characterized as homonymous or bilateral in eight studies [1,23,29,42,82,93,94,100]. For symptom type, negative symptoms predominated in RM in six studies [7,8,20,49,66,87], with occasional positive symptoms, whereas positive symptoms predominated in MA in six studies [1,13,20,28,42,66], with occasional negative symptoms. Duration also varied, with RM ranging from seconds to one hour but potentially prolonged to hours or weeks in eight studies [3,7,8,15,20,49,57,87], compared to MA typically lasting five to 60 minutes and rarely exceeding one hour in seven studies [1,7,20,28,42,66,68]. Regarding frequency, RM was noted as recurrent and variable in four studies [8,20,57,87], while MA was stereotyped in five studies [1,24,28,41,42]. Visual field defects differed notably, with RM most commonly involving central or pericentral areas, along with arcuate, altitudinal, quadrantic, and tunnel vision described in eight studies [3,7,15,16,20,49,57,66], in contrast to MA, which typically featured fortification spectra, scotomas, zigzag lines, and heat waves in seven studies [1,27-29,42,46,82]. Regarding associated headache, it was less common in RM, where it may precede, accompany, or follow visual loss, usually ipsilateral, in four studies [7,15,20,66]. In contrast, in MA, headache is often contralateral and typically follows or accompanies the aura, as evidenced by six studies [1,28,42,43,68,76].

Temporal and Progressive Features

RM symptoms exhibit notable variability in duration and progression, typically ranging from seconds to an hour, though some cases-particularly in RM, extend to hours or even weeks, as reported in multiple studies [3,66,67]. In 78-100% of instances, symptoms resolve in less than one hour, but recurrent episodes may lead to prolonged durations or, rarely, permanent vision loss [15,54,67]. Visual disturbances can occur independently of headache (known as acephalgic RM), or they can precede, accompany, or follow a headache, with intra-individual variability often observed in symptom onset, progression, and overall duration [3,7,15,66,67].

In contrast, MA symptoms are generally more consistent, lasting five to 60 minutes with a gradual spread of visual phenomena in the majority of cases [1,5,6,68]. This duration applies to 75-90% of episodes, with fewer than five minutes in 2-3% and more than 60 minutes in 6-10% [1,5,6,68,69]. Aura typically precedes the headache phase, often with a gap of less than 30 minutes, though intra-individual variability in progression and duration is also commonly reported [1,5,6,14,68].

Overall, temporal features underscore key distinctions: MA tends to be more stereotyped in duration and progression, reliably preceding headache, whereas RM displays greater variability, potentially extending beyond typical limits and showing a less predictable relationship to headache, if headache occurs at all [1,3,5,6]. While acephalgic forms of RM are described in some literature, standard diagnostic criteria often require an associated headache, and underreporting of transient, isolated episodes may contribute to gaps in understanding [3,15,66,67]. The quality of evidence is high for MA, drawn from large-scale studies, but moderate for RM, primarily based on smaller case series and reviews [7,18,66,67].

Associated Symptoms and Comorbidities

RM is less commonly associated with concurrent migraine headache compared to other migraine subtypes, and photophobia is rare; however, recurrent episodes may lead to permanent visual loss in some cases [3,7,15,54,66,67]. It can be linked to vascular pathologies, including retinal hemorrhage, arterial occlusion, or transient ischemia, which underscore its potential for more localized ocular complications rather than widespread neurological involvement [67,70].

MA, by contrast, is frequently accompanied by a broader array of neurological symptoms beyond visual disturbances, such as sensory changes (e.g., tingling or numbness), language difficulties (e.g., aphasia), and motor impairments [71,72]. This condition is primarily attributed to CSD, a wave of neuronal depolarization, and carries a rare but noted increased risk of stroke or cardiovascular events, particularly in individuals with additional risk factors like smoking or oral contraceptive use [73,74].

Both conditions share potential genetic underpinnings, with genes associated with familial hemiplegic migraine (such as CACNA1A, ATP1A2, and SCN1A) implicated in certain aura subtypes, though evidence for genetic influences is more robust in MA due to larger-scale studies [72,75].

In summary, MA tends to manifest with a broader spectrum of neurological symptoms, reflecting its cortical origins, while RM is more tightly linked to vascular ocular issues and the risk of permanent visual impairment [3,7,10,66,67]. Genetic predispositions may play a role in both, but the body of evidence supporting this is stronger for MA, potentially due to its higher prevalence and more extensive research [2,13,18,22,72,75].

Diagnostic Criteria and Clinical Differentiation

Monocular visual loss holds high diagnostic value for RM, with moderate reliability that requires exclusion of other causes, and it clinically implies RM if the symptoms are strictly monocular and reversible [76]. Homonymous or bilateral symptoms have high diagnostic value for MA, along with high reliability, suggesting a cortical origin typical of MA [1,5,14]. Positive visual phenomena also carry high diagnostic value for MA, with high reliability, as they are classic for aura but less common in RM [6,23,71]. Negative visual phenomena offer moderate diagnostic value for RM, with moderate reliability, being common in RM, though they can also occur in aura [3,7,66]. A duration of five to 60 minutes has low diagnostic value for MA and moderate reliability, as it is typical for both conditions, with shorter or longer durations slightly favoring RM [68]. Association with headache presents variable diagnostic value and moderate reliability, since both conditions can involve headache but with differing timing [1,3,5,6,14,17,66,67]. Finally, the ICHD-3 criteria provide high diagnostic value and high reliability, serving as the standard for diagnosis, though some propose modifications specifically for RM. Table 3 summarizes the distinguishing clinical features and implications drawn from this review.

**Table 3:** Distinguishing clinical features and implications of RM compared to MA For monocular visual loss [3,7,8,15,20,49,57,66]; homonymous/bilateral symptoms [1,23,29,42,82,93,94,100]; positive visual phenomena [1,13,20,28,42,66]; negative visual phenomena [7,8,20,49,66,82]; duration: five to 60 minutes [1,3,7,8,15,20,28,42,49,57,66,68,87]; association with headache [1,28,42,43,68,76]; ICHD-3 criteria [1,3,7,13,18,28,42,49,66,67] ICHD: International Classification of Headache Disorders; MA: migraine with aura; RM: retinal migraine

Clinical feature	Diagnostic value	Reliability	Clinical implications
Monocular visual loss	High for RM	Moderate (requires exclusion of other causes)	Suggests RM if strictly monocular and reversible
Homonymous/bilateral symptoms	High for MA	High	Suggests cortical origin (MA)
Positive visual phenomena	High for MA	High	Classic for aura; less common in RM
Negative visual phenomena	Moderate for RM	Moderate	Common in RM, but can occur in aura
Duration: five to 60 minutes	Low for MA	Moderate	Typical for both; shorter or longer slightly favors RM
Association with headache	Variable	Moderate	Both can be associated; timing differs
ICHD-3 criteria	High	High	Standard for diagnosis; some propose modifications for RM

Pathophysiological Mechanisms

RM is primarily hypothesized to involve vascular dysregulation, including vasospasm or transient ischemia affecting the retinal or ocular vasculature, leading to temporary monocular vision disturbances such as scotomas or blindness [3,7,8,15,20,49,66,67,86,89]. In rare instances, recurrent episodes may culminate in permanent visual loss due to retinal infarction, potentially exacerbated by underlying vascular pathologies like arterial occlusion or hemorrhage [7,15,20,50,66,87,101]. Emerging theories also suggest contributions from retinal spreading depression or the release of neuropeptides such as calcitonin gene-related peptide, though the exact mechanisms remain incompletely understood due to the condition's rarity [2,7,15,20,66,86,95].

MA is fundamentally linked to CSD, a slow-propagating wave of neuronal and glial depolarization across the cerebral cortex that disrupts normal brain function and manifests as transient focal neurological symptoms [2,56,74]. Genetic factors play a significant role, with mutations in genes like CACNA1A, ATP1A2, and SCN1A implicated in familial hemiplegic migraine, a rare subtype, and potentially contributing to broader aura susceptibility through ion channel dysfunction [13,75]. Additionally, MA is associated with an elevated risk of cardiovascular and cerebrovascular events, such as stroke, likely due to shared vascular risk factors and endothelial dysfunction [10,19].

In summary, the pathophysiology of RM centers on vascular mechanisms localized to the eye, contrasting with the cortical neuronal processes underlying MA [2,3,7,8,49,56,67,74]. Genetic predispositions are more firmly established for MA, especially in its familial forms, supported by extensive genetic studies, whereas evidence for RM remains limited [2,3,7,13,15,75]. A distinctive concern in ocular or RM is the potential for permanent visual impairment, highlighting the need for prompt evaluation to rule out serious vascular complications [15,20,44,50,66,85,87,101].

Comparative analysis

Distinguishing Features

Distinguishing features between RM and MA include laterality, where RM is classically monocular, affecting vision in only one eye [3,7,15], whereas MA is typically binocular, impacting both eyes [8,20,49,54]. Regarding the risk of permanent vision loss, RM, particularly its retinal subtype, may progress to permanent monocular visual loss in recurrent cases due to potential vascular complications like infarction, while no such risk was identified for MA in the reviewed literature [15,20,50,66,87,101]. In terms of association with headache, both conditions can involve headache, but RM often manifests as an isolated visual symptom without an accompanying headache (known as acephalgic RM), or the headache may precede, coincide with, or follow the visual disturbance, contrasting with MA, where visual symptoms typically precede the headache phase [8,43,67,68,80,89,99]. Table 4 provides a comparative overview of key visual symptom features in RM and MA.

**Table 4:** Comparative overview of key visual symptom features in RM and MA For laterality: RM [3,7,8,15,20,49,57,66], MA [1,23,29,42,82,93,94,100]; symptom type: RM [7,8,20,49,66,82], MA [1,13,20,28,42,66]; duration: RM [3,7,8,15,20,49,57,87], MA [1,7,20,28,42,66,68]; frequency: RM [8,20,57,87], MA [1,24,28,41,42]; visual field defects: RM [3,7,15,16,20,49,57,66], MA [1,27-29,42,46,82]; associated headache: RM [7,15,20,66], MA [1,28,42,43,68,76] MA: migraine with aura; RM: retinal migraine

Feature	RM	MA	Distinguishing characteristics
Laterality	Predominantly monocular (one eye)	Typically homonymous (same side of both eyes), often bilateral	In the referenced studies, RM was described as almost exclusively monocular; MA was rarely monocular and usually homonymous
Symptom type	Negative (scotoma, blindness, visual field loss), sometimes positive (flashing lights, zigzag lines)	Positive (flashing lights, zigzag lines, fortification spectra), sometimes negative (scotoma)	Positive symptoms predominate in aura; negative symptoms predominate in RM
Duration	Seconds to one hour; can be prolonged (hours to weeks)	Five to 60 minutes (typical), rarely more than one hour	RM can be shorter or much longer; aura is stereotyped in duration
Frequency	Recurrent, variable (from less than yearly to weekly)	Recurrent, often stereotyped for individual	Both can be recurrent, but aura is more stereotyped
Visual field defects	Most commonly central or pericentral; arcuate, altitudinal, quadrantic, and tunnel vision also described	Fortification spectra, scotoma, zigzag lines, “heat waves”	RM: more variable, often central or pericentral; aura: more classic patterns
Associated headache	Less common; may precede, accompany, or follow visual loss; usually ipsilateral	Typically follows or accompanies aura, often contralateral	RM: variable timing; aura: headache usually follows aura

Overlapping Characteristics

Overlapping characteristics between RM and MA include shared positive and negative visual phenomena, such as flashing lights, zigzag lines, scotomas, and TVL, which can appear in both conditions despite their differing underlying mechanisms [3,7,8,15,16,49,66,67]. Additionally, both typically feature symptoms lasting less than one hour, often with a gradual spread of visual disturbances, contributing to diagnostic challenges when laterality or other distinguishing features are unclear [20,28,42-44,66-68].

Clinical Implications

Clinically, accurate diagnosis of RM versus MA necessitates a thorough patient history, with particular emphasis on laterality (monocular in RM versus binocular or homonymous in aura), symptom duration (more variable and potentially prolonged in RM compared to the typical five to 60 minutes in aura), and associated features such as headache timing or additional neurological symptoms [7,28,49,54,57,71,76,85-87,89]. For management, while both conditions may benefit from standard migraine therapies like triptans or preventive medications, RM warrants more aggressive prophylactic approaches, such as antiplatelet agents, calcium channel blockers, or close monitoring, in recurrent cases to mitigate the risk of permanent vision loss from vascular complications [3,15,20,44,77,85,92].

Discussion

The evidence base showed that the included studies provided robust findings for MA, supported by large epidemiological and clinical investigations [1,10,18,20,26-28,56,66-68,72,81,90,94], whereas the evidence for ocular/RM was more limited and heterogeneous [3,7,15,16,44,45,74,78]. RM was characterized by typically monocular, often negative visual symptoms of variable duration, rarely leading to permanent visual loss [8,15,20,49,50,54,57,85-87,89,101], while MA was predominantly associated with positive, homonymous, or bilateral symptoms that followed a stereotyped temporal pattern and were often accompanied by additional neurological features [1,13,24,40-43,46,74,82,99]. Regarding diagnostic criteria, the ICHD-3 was widely used, although ongoing debate remains about the classification of RM, particularly in cases involving irreversible visual loss [1,3,7,14,41-43,81]. In terms of pathophysiological mechanisms, vascular dysregulation appeared central to RM, whereas CSD was central to MA [2,8,15,20,49,74]. Finally, both conditions may involve genetic predispositions and are associated with an increased risk of vascular events, but the evidence supporting this link was stronger for MA [10,13,75].

Strengths

This systematic review has several notable strengths that enhance its contributions to the field. First, it adhered rigorously to PRISMA guidelines, including pre-registration on PROSPERO and a comprehensive search across multiple databases (PubMed, Google Scholar, Web of Science, and Scopus), supplemented by LLM-assisted screening of over 126 million academic papers from Semantic Scholar to ensure broad coverage and minimize missed studies. Second, the inclusion of recent publications up to July 2025 captures contemporary insights, such as evolving diagnostic criteria and pathophysiological understandings, providing an up-to-date synthesis. Third, the qualitative narrative approach effectively integrated heterogeneous data from diverse study designs, yielding clinically actionable differentiators (e.g., via tables summarizing symptom prevalences and features) that can aid in reducing diagnostic ambiguity between RM and MA. Finally, the use of adapted JBI tools for quality assessment and multi-step validation in data extraction bolsters the reliability of findings, particularly in a resource-constrained single-author context.

Limitations

This systematic review has several limitations that should be considered when interpreting its findings. These can be broadly grouped into methodological issues and evidence-based concerns.

Methodological issues: The high heterogeneity in study designs, diagnostic criteria, and reporting of visual symptoms precluded a quantitative meta-analysis, limiting the review to a qualitative narrative synthesis. While this approach allowed for thematic integration, it may have amplified subjectivity in interpreting overlaps and distinctions. Additionally, although adapted JBI tools were used for quality assessment, no formal risk-of-bias tool was applied across all studies due to design variability, potentially overlooking systematic biases such as recall bias in retrospective case series or publication bias favoring positive findings. Reliance on an LLM for initial screening of over 126 million papers and data extraction from included studies introduces potential inaccuracies. Although the author manually reviewed and refined selections, LLMs may misinterpret nuanced clinical descriptions or quantitative data, especially in older or less standardized publications. Human oversight mitigated this to some extent, but residual errors in symptom frequency aggregation or classification cannot be entirely ruled out.

As a single-author review, the data extraction process deviated from the standard practice of dual independent extractors with a third resolver for discrepancies, which could increase the risk of extraction bias or errors. To address this, a multi-step validation was implemented, including manual verification of all extracted data against original full texts, random double-checking of 20% of entries (n = 13 studies) with a one-week interval (achieving 95% intra-rater agreement), and cross-referencing of quantitative elements across multiple paper sections. While this aligns with PRISMA guidance for resource-constrained single-author settings, it may not fully replicate the rigor of multi-reviewer extraction. The search strategy was restricted to English-language publications from peer-reviewed sources, excluding non-English studies and gray literature, which may have omitted relevant international or unpublished data, particularly from regions with higher RM prevalence. The temporal scope (1985-2025) captured recent advancements but may have missed foundational works predating 1985.

Evidence-based concerns: The evidence base for RM is notably limited and heterogeneous compared to that for MA. Data on RM were primarily derived from small case series and narrative reviews, with median sample sizes of only 46 patients, reflecting the condition's rarity and diagnostic challenges. In contrast, MA studies often involved large prospective cohorts, leading to more robust epidemiological insights. This disparity may introduce selection bias and reduce the generalizability of comparisons, particularly for RM, where underreporting of acephalgic (headache-free) cases and variability in diagnostic application could skew symptom prevalence estimates. Inherent biases in the primary studies, such as subjective patient reporting of transient visual symptoms and small sample sizes for RM, could inflate estimates of certain features (e.g., monocular laterality) while underestimating rarer complications like permanent vision loss.

These limitations highlight the need for caution in applying the review's conclusions clinically and underscore opportunities for future research, including standardized prospective studies on RM and advanced analytical methods to address heterogeneity.

## Conclusions

This systematic review elucidates the fundamental differences between RM and MA, highlighting the monocular, predominantly negative visual symptoms and variable duration driven by vascular mechanisms in the former versus the bilateral, positive phenomena with consistent cortical patterns in the latter. The evidence for MA is notably stronger, bolstered by extensive epidemiological studies. In contrast, data on RM remain sparse and reliant on case reports, underscoring the need for more rigorous research. Ultimately, advancing diagnostic precision through updated criteria that account for reversibility debates and overlooked acephalgic presentations will be vital to mitigate risks like permanent vision loss and improve patient outcomes.

## Appendices

Appendix A: Detailed large language model-assisted search process

Due to the challenges in identifying standardized data from the literature on these largely subjective and temporary visual symptoms, in addition to the systematic search conducted by the author, a large language model was utilized to search across over 126 million academic papers from the Semantic Scholar corpus. This automated approach aimed to screen a comprehensive list of sources meeting specific inclusion criteria: studies examining adult human patients (aged ≥18 years) diagnosed with ocular migraine and/or migraine with aura, with clear differentiation between these conditions; reports focusing on specific and clearly defined visual symptoms, with differentiation between retinal migraine and migraine aura as a primary or significant emphasis (not exclusively non-visual symptoms); inclusion of frequency or prevalence data on visual symptoms; and primary research studies (observational, cohort, or case-control), systematic reviews/meta-analyses involving ≥10 patients, or case series/reports. The large language model retrieved 500 papers identified as relevant to the search query, sorted by relevance ranking. All sources from the author's manual search were included in these automated results and subsequently discarded to ensure no preferential treatment in the systematic exclusion process. The top results were then sequentially reviewed holistically by the author for inclusion and quality-based filter refinement, resulting in a total of 171 papers screened for further assessment against eligibility criteria.

Appendix B: Detailed instructions for large language model-assisted data extraction

To ensure consistency and accuracy in data extraction, the large language model (LLM) was provided with specific, column-by-column instructions as follows:

Study Design Column

Identify the type of study design used, specifying whether it was observational (such as cross-sectional, cohort, or case-control), a case series, a case report, or a retrospective or prospective analysis. This information was to be sourced primarily from the methods section for explicit descriptions; if not clearly stated, the model was to infer from the study's methodology and data collection approach, listing all applicable design elements if multiple were present.

Visual Symptoms in Retinal Migraine Column

List all visual symptoms reported specifically for retinal migraine, including whether they were monocular or binocular, the type of visual disturbance (such as partial or complete visual loss, or flashing lights), the duration of visual symptoms, and the frequency of symptoms. Emphasis was placed on carefully reviewing the results and discussion sections, prioritizing symptoms with quantitative data on frequency, noting variations if multiple studies reported different findings, and using exact percentages or frequencies when available.

Visual Symptoms in Migraine with Aura Column

List all visual symptoms reported for migraine with aura, categorizing them as positive or negative visual symptoms, providing specific symptom descriptions (such as zigzag lines or colored spots), and including details on the duration and frequency of symptoms. Extraction focused on the results and discussion sections, with priority given to quantitative data, inclusion of multiple descriptions if variations existed, and use of precise percentages or frequencies where possible.

Diagnostic Criteria and Classification Column

Identify the specific diagnostic criteria used, including the diagnostic classification system (such as ICHD-2 or ICHD-3), the specific diagnostic criteria applied, and any modifications to standard diagnostic criteria. This information was to be located in the methods, introduction, or dedicated diagnostic criteria sections, with all referenced classification systems noted if multiple were mentioned, and specific modifications described if criteria were partially altered.

Frequency of Symptoms in Ocular (Retinal) Migraine Column

Defined as the percentage of the study cohort with retinal migraine who experienced a particular visual symptom.

Frequency of Symptoms in Migraine Aura Column

Similarly captured the percentage of the study cohort with migraine aura who experienced a particular visual symptom.
